# Association between baseline smoking status and clinical outcomes following myocardial infarction

**DOI:** 10.3389/fcvm.2022.918033

**Published:** 2022-07-22

**Authors:** Seok Oh, Ju Han Kim, Kyung Hoon Cho, Min Chul Kim, Doo Sun Sim, Young Joon Hong, Youngkeun Ahn, Myung Ho Jeong

**Affiliations:** ^1^Department of Cardiology, Chonnam National University Hospital, Gwangju, South Korea; ^2^Department of Cardiology, Chonnam National University Medical School, Hwasun, South Korea

**Keywords:** myocardial infarction, treatment outcome, coronary artery disease, smoking, ischemic heart disease

## Abstract

**Background:**

Whether the effect of smoking on clinical outcomes following an acute myocardial infarction (AMI) is beneficial or detrimental remains inconclusive. We invesetigated the effect of smoking on the clinical outcomes in patients following an AMI.

**Methods:**

Among 13,104 patients between November 2011 and June 2015 from a nationwide Korean AMI registry, a total of 10,193 participants were extracted then classified into two groups according to their smoking habit: (1) smoking group (*n* = 6,261) and (2) non-smoking group (*n* = 3,932). The participants who smoked were further subclassified according to their smoking intensity quantified by pack years (PYs): (1) <20 PYs (*n* = 1,695); (2) 20–40 PYs (*n* = 3,018); and (3) ≥40 PYs (*n* = 2,048). Each group was compared to each other according to treatment outcomes. The primary outcome was the incidence of major adverse cardiac and cerebrovascular events (MACCEs), which is a composite of all-cause mortality, non-fatal MI (NFMI), any revascularization, cerebrovascular accident, rehospitalization, and stent thrombosis. Secondary outcomes included the individual components of MACCEs. The Cox proportional hazard regression method was used to evaluate associations between baseline smoking and clinical outcomes following an AMI. Two propensity score weighting methods were performed to adjust for confounders, including propensity score matching and inverse probability of treatment weighting.

**Results:**

While the incidence of all clinical outcomes, except for stent thrombosis, was lower in the smoking group than in the non-smoking group in the unadjusted data, the covariates-adjusted data showed statistical attenuation of these differences but a higher all-cause mortality in the smoking group. For smokers, the incidence of MACCEs, all-cause mortality, cardiac and non-cardiac death, and rehospitalization was significantly different between the groups, with the highest rates of MACCE, all-cause mortality, non-cardiac death, and rehospitalization in the group with the highest smoking intensity. These differences were statistically attenuated in the covariates-adjusted data, except for MACCEs, all-cause mortality, and non-cardiac death, which had the highest incidence in the group with ≥40 PYs.

**Conclusion:**

Smoking had no beneficial effect on the clinical outcomes following an AMI. Moreover, for those who smoked, clinical outcomes tended to deteriorate as smoking intensity increased.

## Introduction

Cardiovascular disease (CVD) is one of the leading causes of mortality worldwide, responsible for approximately 32% of all global mortalities (1–3). Among the CVDs, acute myocardial infarction (AMI) leads to a substantial 30-day mortality of 3–14% (4).

Tobacco use, which is one of the most important but primary avoidable risk factor responsible for diverse CVDs such as AMI and stroke (5–8), accounts for about 11% of global cardiovascular deaths (9). However, numerous clinical studies have demonstrated that smokers might have more favorable outcomes post-AMI than non-smokers (10, 11). The term “smoker’s paradox” was coined to explain the characteristic findings from observational studies where the short-term mortality post-AMI tended to be more favorable for smokers than for non-smokers (12). According to a clinical study conducted by Symons et al. (11), tobacco use seemed to be a negative predictor of post-AMI remodeling of the left ventricle, which was consistent with the term “smoker’s paradox.” Among patients with coronary artery disease (CAD) who underwent percutaneous coronary intervention (PCI), a large single-center study conducted in China reported similar 2-year outcomes between persistent smokers and those who never smoked (13). In a Taiwanese study, this “smoker’s paradox” tended to extend long-term outcomes in patients with stable CAD who received PCI (14).

Nevertheless, many recent studies have demonstrated clinical results negating these paradoxical findings. Liu et al. published the first follow-up report on the long-term effects of persistent smoking in Chinese male patients after stent implantation, which showed that poor adherence to smoking cessation is associated with a high incidence of all-cause mortality and major adverse cardiac and cerebrovascular events (MACCEs) (15). In a clinical study based on the SYNTAX (SYNergy Between PCI With TAXUS and Cardiac Surgery) trial (16), smoking was associated with an increased risk of MACCE and the composite outcome of mortality, myocardial infarction (MI), and stroke at the 5-year follow-up. In a Singaporean cohort study, the seemingly beneficial effects of smoking on the 30-day and 1-year mortalities disappeared after adjustment, whereas the risk of recurrent MI was significantly higher in smokers with ST-segment elevation MI (STEMI) and non-STEMI (NSTEMI) (17), which confirmed the smoker’s pseudo-paradox for mortality.

It remains inconclusive whether the effect of smoking on clinical outcomes following an AMI is beneficial or detrimental. Moreover, there has been a paucity of information on the dose–response relationship between tobacco use and clinical outcomes in patients with AMI. To fill these gaps, the present study aimed to investigate the effects of smoking on clinical outcomes in these patients.

## Materials and methods

### Study scheme, setting, and population

**FIGURE 1:**
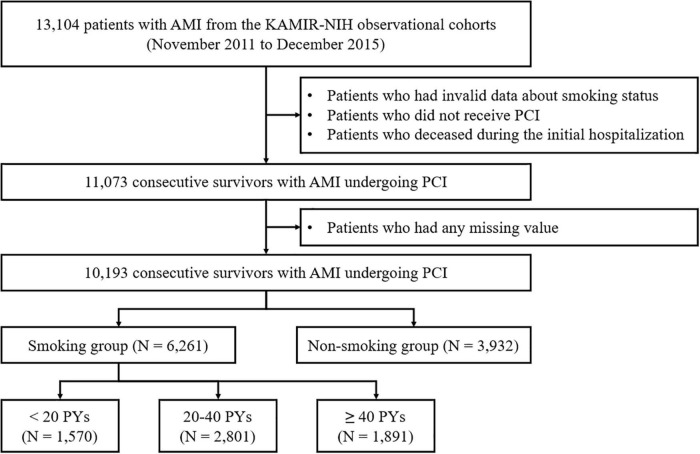
Flow chart of the study participants. AMI, acute myocardial infarction; KAMIR-NIH, Korea acute myocardial infarction Registry-National Institutes of Health; PCI, percutaneous coronary intervention; PY, pack year.

The study scheme is illustrated in Figure 1. All patient data were extracted from the Korea Acute Myocardial Infarction Registry-National Institutes of Health (KAMIR-NIH) registry, which is a nationwide, multicenter-based, and online-based observational cohort registry. The KAMIR-NIH registry has collected clinical data on patients with AMI from 20 PCI-capable tertiary cardiovascular institutions from November 2011 to December 2015. This registry harbors all clinical information on the characteristics and treatment outcomes of AMI among the Korean population. The registry protocol was approved by the ethics committee of each participating institution and was then published (18).

Among 13,104 patients from the KAMIR-NIH cohort, we initially screened 11,073 patients after excluding (a) patients who had invalid data regarding their smoking status; (b) patients who did not receive PCI; and (c) patients who died during the initial hospitalization. After excluding patients who had any missing data, 10,193 consecutive participants were finally selected. They patients were classified into two groups according to their smoking habit: (a) smoking group (*n* = 6,261) and (b) non-smoking group (*n* = 3,932). Moreover, the smoking group was further subdivided into three groups according to the smoking intensity quantified by pack years (PYs): (a) <20 PYs (*n* = 1,695); (b) 20–40 PYs (*n* = 3,018); and (c) ≥40 PYs (*n* = 2,048).

### Definitions

In accordance with contemporary guidelines, AMI was defined as myocardial necrosis with a change (rise or fall) in cardiac markers and MI-associated findings, including at least one of the following (19, 20): (a) MI-related clinical symptoms; (b) new findings in an electrocardiogram (ECG) suggestive of an MI such as T-wave inversion or ST-segment deviation; (c) development of pathological Q-waves on an ECG; (d) definite evidence of loss of myocardial viability or abnormalities in the regional wall motion from cardiovascular imaging modalities; and (e) presence of an angiographically confirmed intracoronary thrombus. STEMI was diagnosed as a new-onset ST elevation in >2 continuous leads (>0.2 mV in leads V1–3 or >0.1 mV in all other leads on a 12-lead surface ECG) (18). Image-guided PCI was defined as the utilization of intravascular ultrasound or optical coherence tomography during the index PCI procedure. Left main coronary artery (LMCA) disease was determined as a ≥50% reduction in the LMCA diameter. Multivessel disease (MVD) refers to significant coronary stenosis in ≥2 epicardial coronary arteries (≥70% stenosis in ≥2 epicardial coronary arteries or ≥70% stenosis in one epicardial coronary artery, with ≥50% stenosis of the LMCA). The degree of intracoronary flow was estimated according to the Thrombolysis In Myocardial Infarction (TIMI) flow grade. As a central measure of left ventricular (LV) systolic function, LV ejection fraction (LVEF) was measured and quantified by transthoracic echocardiography. An infarct-related artery (IRA) was defined as an epicardial coronary artery where the atherothrombotic plaques ruptured, leading to an AMI. Lesion characteristics were stratified according to the American College of Cardiology/the American Heart Association (ACC/AHA) classification of coronary lesions.

We analyzed several parameters as indicators of treatment delay for a PCI: (a) total ischemic time (TIT), (b) symptom-to-door time (S2DT), and (c) door-to-balloon time (D2BT). S2DT was defined as the interval from the onset of symptoms to hospital presentation. D2BT was defined as the interval from hospital presentation to when PCI ballooning was performed. TIT was derived as the sum of S2DT and D2BT.

### Information on clinical and procedural characteristics

The baseline clinical and procedural characteristics were analyzed. Information on the prescribed medications was also collected. The baseline clinical characteristics included age, gender, utilization of emergency medical service (EMS), Killip functional class, parameters for pre-hospital and in-hospital treatment delay (TIT, S2DT, and D2BT), body mass index (BMI), serum creatinine level, previous medical history (hypertension, diabetes mellitus, dyslipidemia, prior ischemic heart disease [IHD], prior heart failure, and prior cerebrovascular accident [CVA]), family history of IHD, use of thrombolytics, final diagnosis (STEMI or NSTEMI), and LVEF. The angiographic and procedural characteristics included the anatomical site of vascular access (femoral approach vs. radial approach), use of glycoprotein IIb/IIIa (GPIIb/IIIa) inhibitors, use of thrombus aspiration, use of image-guided PCI, IRA (LMCA or left anterior descending coronary artery [LAD] vs. left circumflex coronary artery [LCX] or right coronary artery [RCA]), lesion characteristics, preprocedural TIMI flow grade, and presence of LMCA disease or MVD. The discharge medications included information on the use of aspirin, P2Y12 inhibitors, beta-blockers, angiotensin-converting enzyme inhibitors (ACEi) or angiotensin receptor blockers (ARB), and statins.

The smoking behavior of each participant was secured by history taking at admission. If impossible to secure patient’s self-reported data on smoking habit, researchers referred to family member’s statements.

### Clinical outcomes

The primary endpoint was the occurrence of MACCEs. A MACCE was defined as the composition of all-cause mortality, non-fatal MI (NFMI), any revascularization, CVA, rehospitalization, and stent thrombosis. The secondary endpoints were all-cause mortality, NFMI, any revascularization, CVA, rehospitalization, and stent thrombosis. Rehospitalization was defined as any admission due to angina pectoris or heart failure. Each component of clinical outcomes was secured through patient or family self-report in the outpatient setting, or all available electronic medical records in the inpatient setting.

### Statistical analysis

Statistical analysis was performed to evaluate the differences in the clinical outcomes between the groups. It was performed using both STATA version 15.0 (StataCorp, College Station, TX, United States) and SPSS version 25.0 (SPSS Inc., Armonk, NY, United States). Discrete variables are expressed as numbers with percentages, whereas continuous variables are expressed as medians with interquartile ranges. In the comparative analysis, discrete variables were analyzed using Pearson’s chi-square test, Fisher’s two-by-two exact test, or the Mantel–Haenszel linear-by-linear association, while continuous variables were analyzed using Student’s *t*-test, Mann–Whitney test, and the analysis of variance test. All results were rendered statistically significant at *p* < 0.05.

The treatment estimates over time between the groups were compared using the survival analysis. We plotted the Kaplan–Meier survival curves stratified according to the baseline smoking status. We applied the Cox proportional hazard regression method for multivariable analysis. To minimize selection bias and adjust for group-by-group differences, we applied two different statistical methods including propensity score matching (PSM) and the inverse probability of treatment weighting (IPTW), to determine whether smoking really affected the clinical outcomes in patents with AMI. The propensity scores were computed with the following 32 covariates in the overall study population: sex (male vs. female), age (≥75 vs. <75 years), S2DT (≥4 h vs. <4 h), D2BT (≥90 min vs. <90 min), EMS utilization, Killip functional class (III–IV vs. I–II), BMI (≥25 kg/m^2^ vs. <25 kg/m^2^), previous history (hypertension, diabetes mellitus, dyslipidemia, prior IHD, prior heart failure, and prior CVA), family history of IHD, serum creatinine level (≥1.5 vs. <1.5 mg/dL), discharge medications (aspirin, P2Y12 inhibitors, beta-blockers, ACEi/ARBs, statins), vascular access (femoral vs. radial approach), GPIIb/IIIa inhibitors use, thrombus aspiration, image-guided PCI, IRA (LMCA or LAD vs. LCX or RCA), ACC/AHA lesion characteristics (B2/C vs. A/B1), preprocedural TIMI flow grade (TIMI 0–I vs. II–III), LMCA disease, MVD, LVEF (≥40% vs. <40%), thrombolysis, and the final diagnosis (STEMI vs. NSTEMI).

### Ethics statement

The study protocol was designed according to the ethical principles of the Declaration of Helsinki and was approved by the Institutional Review Board of Chonnam National University Hospital. Informed consent was waived given the retrospective nature of the study.

## Results

### Baseline clinical and procedural characteristics of the overall population

In the present study, 10,193 consecutive patients were included in the analysis. Among them, 6,261 and 3,932 patients were allocated to the smoking and non-smoking groups, respectively (Figure 1). The baseline characteristics are summarized in Table 1, demonstrating the different tendencies between the groups.

**TABLE 1:** Baseline characteristics of the patients.

Characteristics	Before PSM			After PSM		
	Non-smoking group	Smoking group	*P*-value	Non-smoking group	Smoking group	*P*-value
	(*n* = 3,932)	(*n* = 6,261)		(*n* = 1,647)	(*n* = 1,647)	
Male patients	1,705 (43.4)	6,001 (95.8)	**<0.001**	1,427 (86.6)	1,427 (86.6)	1.000
Age ≥75 years	1,366 (34.7)	793 (12.7)	**<0.001**	297 (18.0)	282 (17.1)	0.492
EMS utilization	562 (14.3)	959 (15.3)	0.158	266 (16.1)	264 (16.0)	0.924
Killip functional class III–IV	516 (13.1)	587 (9.4)	**<0.001**	171 (10.4)	190 (11.5)	0.289
Total ischemic time ≥12 h	2,037 (51.8)	2,709 (43.3)	**<0.001**	747 (45.4)	731 (44.4)	0.575
Total ischemic time, h	13 (3–39)	8 (3–27)	0.185	9 (3–30)	8 (3-31)	0.647
Onset-to-door time ≥4 h	2,069 (52.6)	2,714 (43.4)	**<0.001**	743 (45.1)	755 (45.8)	0.675
Onset-to-door time, h	4 (1–17)	3 (1–10)	0.001	3 (1–11)	3 (1–10)	0.572
Door-to-balloon time ≥90 min	2,225 (56.6)	3,091 (49.4)	**<0.001**	871 (52.9)	867 (52.6)	0.889
Door-to-balloon time, min	144 (61–1033)	88 (56–775)	0.651	112 (58–904)	106 (57–846)	0.697
BMI ≥25 kg/m^2^	1,225 (31.2)	2,428 (38.8)	**<0.001**	597 (36.2)	614 (37.3)	0.539
**Previous history**						
Hypertension	2,402 (61.1)	2,631 (42.0)	**<0.001**	835 (50.7)	856 (52.0)	0.464
Diabetes mellitus	1,253 (31.9)	1,516 (24.2)	**<0.001**	437 (26.5)	487 (29.6)	0.052
Dyslipidemia	454 (11.5)	710 (11.3)	0.750	184 (11.2)	209 (12.7)	0.179
Prior IHD	623 (15.8)	822 (13.1)	**<0.001**	244 (14.8)	261 (15.9)	0.411
Prior heart failure	64 (1.6)	48 (0.8)	**<0.001**	20 (1.2)	15 (0.9)	0.396
Prior CVA	288 (7.3)	316 (5.1)	**<0.001**	91 (5.5)	83 (5.0)	0.533
Family history of IHD	194 (4.9)	497 (7.9)	**<0.001**	101 (6.1)	104 (6.3)	0.829
Serum creatinine ≥1.5 mg/dL	426 (10.8)	455 (7.3)	**<0.001**	135 (8.2)	136 (8.3)	0.949
Use of thrombolysis	29 (0.7)	67 (1.1)	0.091	15 (0.9)	16 (1.0)	0.857
STEMI as a final diagnosis	1,861 (47.3)	3,372 (53.9)	**<0.001**	839 (50.9)	865 (52.5)	0.365
LVEF <40%	530 (13.5)	610 (9.7)	**<0.001**	183 (11.1)	213 (12.9)	0.108
**Procedural characteristics**						
Femoral approach	2,512 (63.9)	3,856 (61.6)	**0.020**	1,038 (63.0)	1,058 (64.2)	0.469
GPIIb/IIIa inhibitors	534 (13.6)	1,045 (16.7)	**<0.001**	254 (15.4)	249 (15.1)	0.809
Thrombus aspiration	864 (22.0)	1,712 (27.3)	**<0.001**	411 (24.9)	435 (26.4)	0.338
Image-guided PCI	792 (20.1)	1,447 (23.1)	**<0.001**	369 (22.4)	403 (24.5)	0.162
Infarct-related artery			**<0.001**			0.153
LMCA or LAD	2,008 (51.1)	2,961 (47.3)		823 (50.0)	782 (47.5)	
Others (LCX or RCA)	1,924 (48.9)	3,300 (52.7)		524 (50.0)	865 (52.5)	
ACC/AHA lesion B2/C	3,364 (85.5)	5,519 (88.1)	**<0.001**	1,443 (87.6)	1,423 (86.4)	0.300
Preprocedural TIMI flow 0–I	2,225 (56.6)	3,640 (58.1)	0.123	983 (59.7)	935 (56.8)	0.090
LMCA disease	180 (4.6)	256 (4.1)	0.235	70 (4.2)	78 (4.7)	0.501
Multivessel disease	2,051 (52.2)	3,017 (48.2)	**<0.001**	820 (49.8)	851 (51.7)	0.280
**Discharge medications**						
Aspirin	3,930 (99.9)	6,253 (99.9)	0.335	1,646 (99.9)	1,647 (100.0)	1.000
P2Y12 inhibitors	3,925 (99.8)	6,249 (99.8)	0.877	1,644 (99.8)	1,642 (99.7)	0.726
Beta-blockers	3,392 (86.3)	5,440 (86.9)	0.370	1,429 (86.8)	1,404 (85.2)	0.209
ACE inhibitors or ARBs	1,589 (82.5)	1,569 (81.5)	0.402	1,369 (83.1)	1,339 (81.3)	0.172
Statins	1,815 (94.2)	1,809 (93.9)	0.682	1,569 (95.3)	1,553 (94.3)	0.210

Values are presented as number (percentage) for categorical values and median (interquartile range) for continuous variables. ACC, the American College of Cardiology; ACE, angiotensin-converting enzyme; AHA, American Heart Association; ARB, angiotensin receptor blocker; BMI, body-mass index; CVA, cerebrovascular accident; EMS, emergency medical service; GPIIb/IIIa, glycoprotein IIb/IIIa; IHD, ischemic heart disease; LAD, left anterior descending coronary artery; LCX, left circumflex coronary artery; LMCA, left main coronary artery; LVEF, left ventricular ejection fraction; PCI, percutaneous coronary intervention; PSM, propensity score matching; RCA, right coronary artery; STEMI, ST-segment elevation myocardial infarction; TIMI, Thrombolysis In Myocardial Infarction.

All bold values denote statistical significance at the p < 0.05.

The smoking group was younger and had a higher proportion of males and obese patients; this group was less likely to present with Killip functional class III–IV but more likely to present with STEMI and had less delay in treatment, with shorter TIT, S2DT, and D2BT. Additionally, the smoking group had a lower comorbidity burden, with a lower prevalence of hypertension, diabetes mellitus, prior IHD, prior heart failure, and prior CVA, while having a high proportion of family history of IHD. Patients in this group were more likely to have lower creatinine levels and better LVEF.

During the index procedure, there was a greater percentage of patients who received GPIIb/IIIa inhibitors, thrombus aspiration, and image-guided PCI in the smoking group. Furthermore, the smoking group had a lower proportion of patients with the LMCA or LAD as an IRA, and a higher proportion of ACC/AHA lesions B2/C. However, MVD was more prevalent in the non-smoking group than in the smoking group. Similar findings were noted regarding discharge medications between the two groups. All differences were statistically balanced after adjustment with PSM.

### Baseline clinical and procedural characteristics of the smoking population

Three subgroups in the smoking population, which were stratified by smoking PYs, were also analyzed and are summarized in Table 2. A higher proportion of male patients, Killip functional class III–IV, and increased incidence of diabetes mellitus were noted, whereas the proportion of family history of IHD decreased, as the smoking PYs increased. In terms of age and variables such as TIT, S2DT, BMI, hypertension, serum creatinine level, LVEF, and MVD, the J-curve patterns with the highest or lowest values were found in the group with 20–40 PYs. The group with the highest smoking PYs had the oldest age but the lowest prevalence of obesity. In coronary angiographic procedural characteristics, no significant difference was noted except for MVD, which implies that the ≥40 PY group had the highest proportion of MVD. All differences were statistically balanced after adjustment with IPTW.

**TABLE 2:** Baseline characteristics of the smoking group according to smoking pack years.

Characteristics	Before IPTW				After IPTW			
	<20 PYs	20–40 PYs	≥40 PYs	*P*-value	<20 PYs	20-40 PYs	≥40 PYs	*P*-value
	(*n* = 1,570)	(*n* = 2,800)	(*n* = 1,891)		(*n* = 6,263)	(*n* = 6,261)	(*n* = 6,269)	
Male patients	1,433 (91.3)	2,711 (96.8)	1,857 (98.2)	**<0.001**	6,002 (95.8)	6,001 (95.8)	5,994 (95.6)	0.917
Age ≥75 years	213 (13.6)	281 (10.0)	299 (15.8)	**<0.001**	819 (13.1)	805 (12.9)	825 (13.2)	0.962
EMS utilization	258 (16.4)	417 (14.9)	284 (15.0)	0.363	968 (15.5)	961 (15.3)	980 (15.6)	0.971
Killip functional class III-IV	133 (8.5)	243 (8.7)	211 (11.2)	**0.006**	595 (9.5)	590 (9.4)	596 (9.5)	0.995
Total ischemic time ≥12 h	696 (44.3)	1,177 (42.0)	836 (44.2)	0.208	2,761 (44.1)	2,714 (43.3)	2,639 (42.1)	0.464
Total ischemic time, h	8 (2–28)	7 (2–26)	8 (3–30)	**0.016**	8 (2–28)	8 (3–27)	7 (3–27)	0.461
Onset-to-door time ≥4 h	666 (42.4)	1,170 (41.8)	878 (46.4)	**0.005**	2,706 (43.2)	2,717 (43.4)	2,697 (43.0)	0.974
Onset-to-door time, h	3 (1–10)	3 (1–9)	3 (1–11)	**<0.001**	3 (1–10)	3 (1–9)	3 (1–10)	0.738
Door-to-balloon time ≥90 min	790 (50.3)	1,353 (48.3)	948 (50.1)	0.327	3,073 (49.1)	3,093 (49.4)	3,081 (49.1)	0.981
Door-to-balloon time, min	91 (58–819)	85.5 (55–752)	91 (55–781)	0.072	88 (57–790)	88 (56–780)	87 (54–711)	0.396
BMI ≥25 kg/m^2^	622 (39.6)	1,159 (41.4)	647 (34.2)	**<0.001**	2,433 (38.8)	2,428 (38.8)	2,459 (39.2)	0.949
**Previous history**								
Hypertension	693 (44.1)	1,117 (39.9)	821 (43.4)	**0.008**	2,655 (42.4)	2,630 (42.0)	2,620 (41.8)	0.925
Diabetes mellitus	349 (22.2)	641 (22.9)	526 (27.8)	**<0.001**	1,524 (24.3)	1,5226 (24.4)	1,513 (24.1)	0.981
Dyslipidemia	200 (12.7)	312 (11.1)	198 (10.5)	0.101	709 (11.3)	707 (11.3)	711 (11.3)	0.999
Prior IHD	215 (13.7)	344 (12.3)	263 (13.9)	0.203	826 (13.2)	823 (13.1)	864 (13.8)	0.817
Prior heart failure	13 (0.8)	14 (0.5)	21 (1.1)	0.060	50 (0.8)	52 (0.8)	47 (0.7)	0.954
Prior CVA	88 (5.6)	117 (4.2)	111 (5.9)	**0.017**	324 (5.2)	321 (5.1)	335 (5.3)	0.958
Family history of IHD	146 (9.3)	232 (8.3)	119 (6.3)	**0.003**	499 (8.0)	494 (7.9)	509 (8.1)	0.960
Serum creatinine ≥1.5 mg/dL	119 (7.6)	159 (5.7)	177 (9.4)	**<0.001**	458 (7.3)	459 (7.3)	455 (7.3)	0.997
Use of thrombolysis	16 (1.0)	30 (1.1)	21 (1.1)	0.967	68 (1.1)	67 (1.1)	66 (1.1)	0.998
STEMI as a final diagnosis	841 (53.6)	1,546 (55.2)	985 (52.1)	0.105	3,383 (54.0)	3,378 (54.0)	3,412 (54.4)	0.947
LVEF <40%	154 (9.8)	243 (8.7)	213 (11.3)	**0.014**	613 (9.8)	614 (9.8)	627 (10.0)	0.970
**Procedural characteristics**								
Femoral approach	960 (61.1)	1,730 (61.8)	1,166 (61.7)	0.914	3,860 (61.6)	3,868 (61.8)	3,890 (62.0)	0.962
GPIIb/IIIa inhibitors	256 (16.3)	493 (17.6)	296 (15.6)	0.190	1,043 (16.7)	1,044 (16.7)	1,061 (16.9)	0.968
Thrombus aspiration	432 (27.5)	782 (27.9)	498 (26.3)	0.479	1,730 (27.6)	1,711 (27.3)	1,712 (27.3)	0.969
Image-guided PCI	344 (21.9)	636 (22.7)	467 (24.7)	0.123	1,443 (23.0)	1,452 (23.2)	1,449 (23.1)	0.995
Infarct-related artery				0.414				0.991
LMCA or LAD	765 (48.7)	1,314 (46.9)	882 (46.6)		2,976 (47.5)	2,968 (47.4)	2,986 (47.6)	
Others (LCX or RCA)	805 (51.3)	1,486 (53.1)	1,009 (53.4)		3,287 (52.5)	3,293 (52.6)	3,283 (52.4)	
ACC/AHA lesion B2/C	1,383 (88.1)	2,472 (88.3)	1,664 (88.0)	0.952	5,522 (88.2)	5,516 (88.1)	5,499 (87.7)	0.896
Preprocedural TIMI flow 0-I	899 (57.3)	1,673 (59.8)	1,068 (56.5)	0.060	3,643 (58.2)	3,632 (58.0)	3,623 (57.8)	0.971
LMCA disease	61 (3.9)	103 (3.7)	92 (4.9)	0.118	259 (4.1)	258 (4.1)	249 (4.0)	0.961
Multivessel disease	736 (46.9)	1,304 (46.6)	977 (51.7)	**0.001**	3,034 (48.4)	3,016 (48.2)	2,998 (47.8)	0.927
**Discharge medications**								
Aspirin	1,568 (99.9)	2,797 (99.9)	1,888 (99.8)	0.889	6,256 (99.9)	6,255 (99.9)	6,262 (99.9)	0.997
P2Y12 inhibitors	1.565 (99.7)	2,797 (99.9)	1,887 (99.8)	0.301	6,250 (99.8)	6,251 (99.8)	6,257 (99.8)	0.979
Beta-blockers	1,379 (87.8)	2,446 (87.4)	1,615 (85.4)	0.066	5,438 (86.8)	5,437 (86.8)	5,434 (86.7)	0.985
ACE inhibitors or ARBs	1,281 (81.6)	2,343 (83.7)	1,579 (83.5)	0.180	5,211 (83.2)	5,210 (83.2)	5,193 (82.8)	0.938
Statins	1,513 (96.4)	2,677 (95.6)	1,803 (95.4)	0.309	6,001 (95.8)	5,995 (95.7)	6,013 (95.9)	0.961

Values are presented as number (percentage) for categorical values and median (interquartile range) for continuous variables. ACC, American College of Cardiology; ACE, angiotensin-converting enzyme; AHA, the American Heart Association; ARB, angiotensin receptor blocker; BMI, body-mass index; CVA, cerebrovascular accident; EMS, emergency medical service; GPIIb/IIIa, glycoprotein IIb/IIIa; IHD, ischemic heart disease; IPTW, inverse probability of treatment weighting; LAD, left anterior descending coronary artery; LCX, left circumflex coronary artery; LMCA, left main coronary artery; LVEF, left ventricular ejection fraction; PCI, percutaneous coronary intervention; PY, pack year; RCA, right coronary artery; STEMI, ST-segment elevation myocardial infarction; TIMI, Thrombolysis In Myocardial Infarction.

All bold values denote statistical significance at the p < 0.05.

### The 3-year clinical outcomes

The 3-year clinical outcomes of the overall population are summarized in Table 3. In the unadjusted data, the incidence of all clinical outcomes except for stent thrombosis was lower in the smoking group than in the non-smoking group. Although the PSM-adjusted data showed statistical attenuations of these differences, the all-cause mortality in the smoking group was rather high. Additionally, the smoking group tended to have a higher incidence of non-cardiac death but a lower incidence of any revascularization, albeit without significance. These findings were also well-described in the Kaplan–Meier survival curves (Supplementary Figures 1, 2).

**TABLE 3:** Three-year clinical outcomes in propensity score matched patients.

Outcomes	Non-smoking group (*n* = 3,878)	Smoking group (*n* = 6,197)	Unadjusted analysis		PSM-adjusted analysis	
			HR (95% CI)*^a)^*	*P*-value	HR (95% CI)*^b)^*	*P*-value
MACCE*^c)^*	965 (24.9)	1,094 (17.6)	0.68 (0.63–0.74)	**<0.001**	0.94 (0.80–1.10)	0.422
All-cause mortality	370 (9.5)	366 (5.9)	0.60 (0.52–0.70)	**<0.001**	1.41 (1.07–1.87)	**0.015**
Cardiac death	237 (6.1)	204 (3.3)	0.53 (0.44–0.63)	**<0.001**	1.34 (0.93–1.93)	0.110
Non-cardiac death	133 (3.4)	162 (2.6)	0.74 (0.59–0.94)	**0.012**	1.52 (0.98–2.34)	0.060
NFMI	156 (4.0)	181 (2.9)	0.70 (0.57–0.87)	**0.001**	0.92 (0.63–1.34)	0.664
Any revascularization	390 (10.1)	565 (9.1)	0.88 (0.77–1.00)	**0.048**	0.81 (0.65–1.01)	0.060
CVA	113 (2.9)	109 (1.8)	0.59 (0.45–0.76)	**<0.001**	0.78 (0.48–1.28)	0.334
Rehospitalization	216 (5.6)	169 (2.7)	0.47 (0.39–0.58)	**<0.001**	1.01 (0.70–1.44)	0.972
Stent thrombosis	26 (0.7)	43 (0.7)	1.01 (0.62–1.65)	0.961	1.11 (0.47–2.62)	0.806

Values are presented as percentage (number) for categorical values. CI, confidence interval; CVA, cerebrovascular accident; GPIIb/IIIa, glycoprotein IIb/IIIa; HR, hazard ratio; IHD, ischemic heart disease; LVEF, left ventricular ejection fraction; MACCE, major adverse cardiac and cerebrovascular events; NFMI, non-fatal myocardial infarction; PCI, percutaneous coronary intervention; PSM, propensity score matching; TIMI, Thrombolysis In Myocardial Infarction.

^a)^HR corresponds to the smoking group compared with the non-smoking group.

^b)^Adjusted Cox hazard regression analysis included a variety of clinical variables, including age, sex, EMS utilization, total ischemic time, body-mass index, prior medical history, family history of IHD, creatinine level, use of thrombolysis, final diagnosis, LVEF, femoral approach GPIIb/IIIa inhibitors, thrombus aspiration, image-guided PCI, infarct-related artery, TIMI flow grade, LMCA disease, multivessel disease and discharge medications.

^c)^MACCE is defined as a composite of all-cause mortality, NFMI, any revascularization, cerebrovascular accident, rehospitalization and stent thrombosis. All bold values denote statistical significance at the p < 0.05.

**FIGURE 2:**
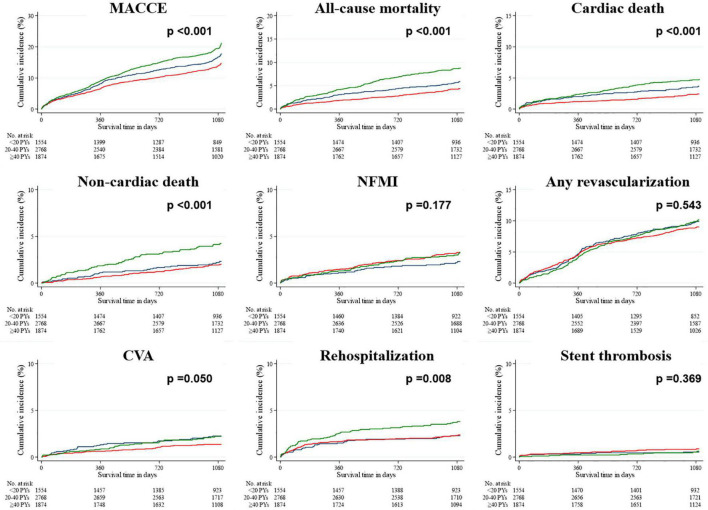
Rates of primary and secondary outcomes for the smoking population after a 3-year follow-up (before IPTW adjustment). The Kaplan–Meier survival curves for cumulative event rates are illustrated according to the baseline smoking status. Blue line indicates the group with <20 PYs. Red line indicates the group with 20–40 PYs. Green line indicates the group with ≥40 PYs. CVA, cerebrovascular accident; IPTW, inverse probability of treatment weighting; MACCE, major adverse cardiac and cerebrovascular event; NFMI, non-fatal myocardial infarction; PY, pack year.

**FIGURE 3:**
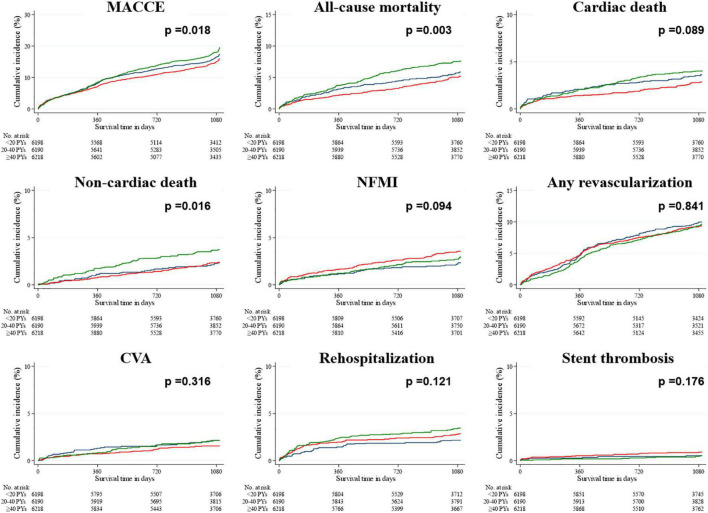
Rates of primary and secondary outcomes for the smoking population after a 3-year follow-up (after IPTW adjustment). The Kaplan–Meier survival curves for cumulative event rates are illustrated according to the baseline smoking status. Blue line indicates the group with <20 PYs. Red line indicates the group with 20–40 PYs. Green line indicates the group with ≥40 PYs. CVA, cerebrovascular accident; IPTW, inverse probability of treatment weighting; MACCE, major adverse cardiac and cerebrovascular event; NFMI, non-fatal myocardial infarction; PY, pack year.

In the unadjusted data within the smoking population (Table 4), the hazard ratios (HRs) of both MACCEs and all-cause mortality were lower and higher in the groups with 20–40 PYs and ≥40 PYs, respectively, than in the group with <20 PYs. The HRs of both cardiac death and CVA were lowest in the group with 20–40 PYs, and that of rehospitalization was highest in the group with ≥40 PYs. After IPTW adjustment, the HRs of all-cause mortality, non-cardiac death, and rehospitalization were highest in the group with ≥40 PYs, whereas the HR of NFMI was highest in the group with 20–40 PYs. In the Kaplan–Meier survival curves (Figures 2, 3), the unadjusted data demonstrated significant differences in MACCEs, all-cause mortality, cardiac death, non-cardiac death, and rehospitalization. These differences were statistically attenuated in the IPTW-adjusted data, except for MACCEs, all-cause mortality, and non-cardiac death.

**TABLE 4:** Three-year clinical outcomes in patients before and after IPTW adjustment.

Outcomes	<20 PYs (*n* = 1,554)	20–40 PYs (*n* = 2,767)	≥40 PYs (*n* = 1,874)		Unadjusted analysis		IPTW-adjusted analysis	
					HR (95% CI)*^a)^*	*P*-value	HR (95% CI)*^b)^*	*P*-value
MACCE*^c)^*	277 (17.8)	409 (14.8)	406 (21.7)	**20–40 PYs**	0.81 (0.70–0.94)	**0.007**	0.90 (0.77–1.05)	0.166
				**≥40 PYs**	1.22 (1.05–1.43)	**0.010**	1.14 (0.97–1.34)	0.099
All-cause mortality	87 (5.6)	116 (4.2)	163 (8.7)	**20–40 PYs**	0.73 (0.56–0.97)	**0.029**	0.87 (0.66–1.16)	0.353
				**≥40 PYs**	1.57 (1.21–2.03)	**0.001**	1.35 (1.03–1.78)	**0.029**
Cardiac death	54 (3.5)	63 (2.3)	87 (4.6)	**20–40 PYs**	0.65 (0.45–0.93)	**0.018**	0.77 (0.53–1.11)	0.163
				**≥40 PYs**	1.35 (0.96–1.90)	0.082	1.15 (0.81–1.64)	0.444
Non-cardiac death	33 (2.1)	53 (1.9)	76 (4.1)	**20–40 PYs**	0.88 (0.57–1.36)	0.564	1.05 (0.67–1.64)	0.842
				**≥40 PYs**	1.92 (1.27–2.88)	**0.002**	1.69 (1.10–2.60)	**0.017**
NFMI	35 (2.2)	89 (3.2)	57 (3.0)	**20–40 PYs**	1.41 (0.95–2.09)	0.084	1.51 (1.01–2.26)	**0.045**
				**≥40 PYs**	1.37 (0.90–2.09)	0.143	1.21 (0.79–1.87)	0.381
Any revascularization	144 (9.3)	245 (8.8)	174 (9.3)	**20–40 PYs**	0.94 (0.76–1.15)	0.532	0.96 (0.78–1.19)	0.707
				**≥40 PYs**	1.01 (0.81–1.26)	0.928	0.94 (0.75–1.18)	0.604
CVA	33 (2.1)	36 (1.3)	40 (2.1)	**20–40 PYs**	0.60 (0.37–0.96)	**0.033**	0.72 (0.44–1.18)	0.193
				**≥40 PYs**	1.02 (0.64–1.61)	0.948	1.04 (0.64–1.69)	0.861
Rehospitalization	35 (2.2)	63 (2.3)	70 (3.7)	**20–40 PYs**	1.00 (0.66–1.51)	0.991	1.27 (0.82–1.95)	0.284
				**≥40 PYs**	1.68 (1.12–2.52)	**0.012**	1.61 (1.04–2.49)	**0.034**
Stent thrombosis	8 (0.5)	25 (0.9)	10 (0.5)	**20–40 PYs**	1.73 (0.78–3.84)	0.177	1.84 (0.81–4.17)	0.145
				**≥40 PYs**	1.05 (0.41–2.65)	0.926	0.90 (0.35–2.34)	0.835

Values are presented as percentage (number) for categorical values. CI, confidence interval; CVA, cerebrovascular accident; GPIIb/IIIa, glycoprotein IIb/IIIa; HR, hazard ratio; IHD, ischemic heart disease; IPTW, inverse probability of treatment weighting; LVEF, left ventricular ejection fraction; MACCE, major adverse cardiac and cerebrovascular events; NFMI, non-fatal myocardial infarction; PCI, percutaneous coronary intervention; TIMI, Thrombolysis In Myocardial Infarction.

^a)^HR corresponds to the each group compared with the reference group (the group with <20 PYs).

^b)^Adjusted Cox hazard regression analysis included a variety of clinical variables, including age, sex, EMS utilization, total ischemic time, body-mass index, prior medical history, family history of IHD, creatinine level, use of thrombolysis, final diagnosis, LVEF, femoral approach GPIIb/IIIa inhibitors, thrombus aspiration, image-guided PCI, infarct-related artery, TIMI flow grade, LMCA disease, multivessel disease and discharge medications.

^c)^MACCE is defined as a composite of all-cause mortality, NFMI, any revascularization, cerebrovascular accident, rehospitalization and stent thrombosis. All bold values denote statistical significance at the p < 0.05.

## Discussion

We compared the 3-year clinical outcomes among patients with AMI based on the baseline smoking status. All clinical information of the 10,193 consecutive patients was analyzed, and 61.4% of the study population were smokers. In this national cohort-based clinical study, the smoking group had lower unadjusted HRs for almost all clinical outcomes, including MACCEs, than did the non-smoking group. Nevertheless, this seemingly beneficial effect of smoking did not exist after PSM adjustment. In the adjusted analysis, all-cause mortality tended to increase in the smoking group compared with that in the non-smoking group.

The “smoker’s paradox” was first mentioned by Weinblatt et al. (21) who demonstrated that smokers had a lower mortality following AMI than non-smokers. This unexpected result was also found in another clinical study (22), which reported a lower prevalence of many CVDs, including AMI, after logistic regression analysis. In this study, the authors have pointed out that the age of smokers was significantly lower than that of non-smokers, and this phenomenon was assumably due to this age difference. As mentioned by Kelly et al. (22), age difference is considered to have mainly contributed to this phenomenon in the present study. Besides, chronological age is one of the key risk factors for developing CVDs *via* various biochemical mechanisms (23–25); it is also related to the different prevalence of comorbid diseases and compositional heterogeneity in several variables related to disease severity, such as MVD or the IRA. In addition, as aforementioned, old age is related to treatment delay due to atypical characteristics of chest symptoms (26). Therefore, when adjusting for confounders such as chronological age, this phenomenon surprisingly disappeared.

Many interesting findings in the baseline clinical characteristics were found. As mentioned earlier, smokers tended to be more often male, younger, and more likely to be obese. Considering evidence of the male predominance regarding smoking (27), our results are sufficiently expected. The finding that the smoker group tended to be younger implies that AMI is more likely to develop at a younger age in this group. Smokers often tend to be more obese, and although still inconclusive, obesity has been known to be associated with favorable outcomes in patients with AMI (28–30). Smokers had lower percentage of other underlying diseases except for dyslipidemia, indicative of a lower burden of co-morbidities. Moreover, smokers were found to have better kidney function, LVEF, and Killip functional class on admission. Impaired kidney function is an adverse prognostic factor in patients with established cardiovascular disorders (31). A low LVEF following AMI is associated with adverse clinical outcomes (32). The Killip classification, a common tool for cardiovascular risk stratification, is also known to be associated with clinical outcomes in patients with AMI (33). There were more patients with STEMI in the smoker group than in the non-smoker group. According to a clinical study based on the KAMIR-NIH registry (34), patients with STEMI tended to have more favorable outcomes than did those with NSTEMI. Smokers had lower TIT, S2DT, and D2BT than non-smokers, indicating that PCI was performed relatively quicker in the smoking group. Higher proportions of female and elderly patients may account for the increase in S2DT in the non-smokers, given that female and elderly patients tend to present with atypical chest pain or no chest pain, which may interfere with the early diagnosis of AMI and contribute to the prolongation of S2DT. Increase in D2BT in the non-smokers can be accounted for by the higher rate of patients with STEMI. All the aforementioned factors are thought to have contributed directly or indirectly to the seemingly better clinical outcomes in the smoker group compared with those in the non-smoker group.

Meanwhile, interesting points in the angiographic procedural characteristics were also noted. It is a generally well-established fact that the affected coronary artery has significant influence on the clinical outcomes of patients with AMI; patients with anterior infarction tend to have worse outcomes following an AMI (35–38). Additionally, patients with coronary MVD are associated with worse outcomes (39). Considering this, non-smokers are expected to have worse prognosis with higher rates of MVD and the LMCA or LAD being an IRA compared with smokers. Nevertheless, we should note that smokers received higher rates of GPIIb/IIIa inhibitors, thrombus aspiration, and image-guided PCI during the procedure. Both GPIIb/IIIa inhibitors and thrombus aspiration are mainly utilized in cases of high burden by the intracoronary thrombus (40, 41). Meanwhile, given that smokers have more complex coronary lesions with a higher prevalence of ACC/AHA B2/C lesions, it seems reasonable to apply image-guided PCI at a higher rate as it has shown to improve outcomes in many randomized clinical trials and observational studies (42–44). It is suggested that smoking certainly accelerates the atherothrombotic process, despite the many advantages mentioned above that smokers have over non-smokers. In fact, there are several clinical studies on the effects of smoking on atherosclerosis in literature review (45–47). Furthermore, it should be noted that both groups were comparable in terms of stent thrombosis, despite decreased unadjusted HRs in the smoker group in all the remaining treatment estimates, which is presumably due to the adverse effect of smoking on atherothrombosis such as plaque disruption (23–25).

We also found that the incidence of any revascularization in the adjusted analysis, although not statistically significant, tended to be relatively low in the smoking group, which is exactly the opposite of the result for all-cause mortality. Intriguingly, several previous studies have reported the results of reduction in the rates of restenosis or target lesion revascularization after interventions (48, 49). However, in recent optical coherence tomography-based studies, tobacco use appears to contribute to neointimal hyperplasia and coronary artery remodeling following PCI, ultimately to be involved in the recurrent event of coronary revascularization (50, 51). As it remains one of the contentious issues, prospective experimental or clinical studies are necessary.

As mentioned earlier, additional analysis was also performed among the smoking groups stratified by smoking intensity. The percentage of males increased along with an increase in PYs (20). Although patients with AMI who smoked tended to be younger than those who did not, the group with the highest smoking PYs seemed to be the oldest among the smoking population. Interestingly, the J-curve phenomenon was observed in age and other variables, including TIT, S2DT, BMI, hypertension, serum creatinine level, LVEF, and MVD. Since atherosclerosis progresses with increasing age, it is plausible that both heart and kidney functions will deteriorate, and the prevalence of hypertension and MVD will increase. Moreover, because elderly patients with AMI tend to present with atypical or no angina, contributing to pre-hospital delay, both S2DT and TIT may be more prolonged in the group with a larger proportion of older-aged patients. Hence, these trends seem to be sufficiently explainable, provided these variables are closely related to age. Unlike both S2DT and TIT, D2BT was similar in the three groups, implicating that the in-hospital treatment delay was statistically equivalent in the 3 groups. Interestingly, although statistically insignificant, the trend toward a low prescription of beta-blockers according to smoking intensity is presumably due to the increase in the prevalence of other comorbid diseases such as chronic obstructive pulmonary disease, which are relative contraindications for beta-blockers.

Owing to these various factors, smoking intensity appeared to influence affect the clinical outcomes. In unadjusted analysis, it seemed to be associated with the incidences of both MACCE and all-cause mortality. In IPTW-adjusted analysis, however, the highest smoking intensity with ≥40 PYs seemed to independently influence the incidences of all-cause mortality, non-cardiac death, and rehospitalization. As a matter of fact, tobacco use can affect the development and prognosis of non-CVDs as well as CVDs; therefore, heavy smokers may be prone to experience a higher rate of non-cardiac death, which may contribute to higher incidence of all-cause mortality. Meanwhile, we should note the treatment outcomes of the group with 20–40 PYs. Although this group showed the lowest rates of MACCE, all-cause mortality, cardiac death, and CVA in unadjusted analysis, it demonstrated the highest incidence of NFMI in the IPTW-adjusted analysis. These paradoxical findings seem to be due to the inclusion of many favorable conditions in the baseline characteristics in this group. In other words, because the group with 20–40 PYs tended to be younger, have shorter TIT, lower prevalence of some comorbidities such as hypertension and CVA, better kidney and heart functions (lower proportions of serum creatinine ≥1.5 mg/dL and LVEF <40%), and a lower incidence of MVD, compared to other two groups, this group seemingly appeared to demonstrate favorable results. After adjusting for all covariates, however, this phenomenon disappeared, showing even poorer outcomes. Since high-intensity tobacco use would facilitate diffuse and advanced coronary atherosclerosis, this result from the covariates-adjusted analysis seems reasonable. Nonetheless, the group with ≥40 PYs (i.e., the group with the heaviest smoking intensity) did not show a significant value of hazard ratio compared to the reference group (the group with <20 PYs). This point, although still not fully explained, may be because patients in this group died of other causes before development or manifestation of NFMI, which consequently masked the harmful effect of heavy smoking. This assumption is supported by the fact that they showed the highest rate of non-cardiac death in both unadjusted and IPTW-adjusted analyses.

Meanwhile, we further investigated a total of 503 patients who deceased during the initial hospitalization (abbreviated as the deceased group) (Supplementary Table 1). The deceased group had different characteristics, compared to their counterpart (i.e., the study population). The deceased group tended to be older; have higher EMS utilization; have poorer clinical manifestations with higher rates of Killip functional class III-IV, and reduced kidney and heart function; be more often diagnosed as STEMI, than the study population. The former had higher prevalence of most comorbidities except for dyslipidemia, compared to the latter. As for the procedural profiles, the deceased group received more femoral approach, GPIIb/IIIa inhibitors, and thrombus aspiration, but less image-guided PCI. The former had relatively high severity of coronary lesions, as supported by the fact that higher rates of ACC/AHA lesion B2/C, preprocedural TIMI flow 0-I, LMCA disease and MVD in this group. Nonetheless, the proportion of patients who currently or previously smoked was relatively low among the deceased group, compared to the study population. It implicates that the majority of in-hospital deaths were caused by high severity of clinical manifestation and high burden of comorbid conditions, not by smoking itself.

Since smoking cessation is well known to be beneficial in terms of mortality risk reduction among patients with acute coronary syndrome (52), many contemporary guidelines emphasize risk factor corrections including smoking cessation (19, 20). According to the clinical practice guideline for cardiac rehabilitation in South Korea (53), the cardiac rehabilitation program includes patient education for secondary prevention such as smoking cessation. It is recommended that interventions for smoking cessation should be given for patient with acute coronary syndrome including AMI who smoke and should be maintained more than 4 weeks. Although many clinical guidelines have emphasized the importance of quitting smoking and the proportion of current smokers among AMI patients decreased from 43.7% in 2005 to 36.1% in 2018 (54), however, only half of patients with AMI in South Korea discontinued tobacco use, as mentioned in a clinical study based on the database of the Korean National Insurance Health Service (55). Hence, the systematic and nationwide efforts to encourage smoking cessation are required in Korea, and the present study will provide objective information on why it is important to discontinue smoking among patients with AMI.

### Study limitations

Although the present study highlighted a pseudo-beneficial effect of tobacco use but a detrimental effect of a high smoking intensity, there are some limitations to be discussed. First, due to the observational but non-randomized nature of the KAMIR-NIH registry, we could not fully establish a causal relationship between the smoking status and clinical outcomes following an AMI. Second, although confounders were adjusted by two propensity score weighting methods in both the overall and smoking population, the problem of selection bias could have persisted due to a variety of reasons, including the data selection by inclusion and exclusion criteria, the presence of missing data and the possibility of unmeasured confounders. Third, the KAMIR-NIH registry included PCI-capable tertiary centers that house and treat high volumes of patients with AMI. This means that this registry did not include patients admitted in small- or medium-sized medical centers. Fourth, the amount of tobacco consumption would be less accurate and less objective because it was obtained by history taking then would depend on the subjective memory of each participant. Moreover, the KAMIR-NIH registry did not contain full detailed information about the exact manner of tobacco consumption before admission and during the follow-up interval. Therefore, these study findings need to be interpreted with caution.

## Conclusion

Although smokers seemingly appeared to have better clinical outcomes following an AMI, these beneficial effects disappeared, and the all-cause mortality was higher in the smoking group upon adjustment. Moreover, within the smoking population, clinical outcomes tended to deteriorate as smoking intensity increased. These results confirm that the “smoker’s paradox” is fictitious and explain why we should help patients with AMI quit tobacco smoking.

## Data availability statement

The raw data supporting the conclusions of this article will be made available by the authors, without undue reservation.

## Ethics statement

The studies involving human participants were reviewed and approved by Institutional Review Board of Chonnam National University Hospital. Written informed consent for participation was not required for this study in accordance with the national legislation and the institutional requirements.

## Author contributions

SO conceptualized the work, carried out software processes, and wrote the original draft of this manuscript. SO, KC, MK, DS, and YH curated the data. SO performed the formal analysis. SO and JK performed the investigation. KC, MK, DS, YH, JK, YA, and MJ edited and reviewed the manuscript. All authors contributed to the article and approved the submitted version.

## Conflict of interest

The authors declare that the research was conducted in the absence of any commercial or financial relationships that could be construed as a potential conflict of interest.

## Publisher’s note

All claims expressed in this article are solely those of the authors and do not necessarily represent those of their affiliated organizations, or those of the publisher, the editors and the reviewers. Any product that may be evaluated in this article, or claim that may be made by its manufacturer, is not guaranteed or endorsed by the publisher.

## Abbreviations

AMI: Acute myocardial infarction
BMI: Body-mass index
CAD: Coronary artery disease
CVD: Cerebrovascular accident
D2BT: Door-to-balloon time
ECG: Electrocardiogram
EMS: Emergency medical service
HR: Hazard ratio
IHD: Ischemic heart disease
IPTW: Inverse probability of treatment weighting
IRA: Infarct-related artery
KAMIR-NIH: Korea Acute Myocardial Infarction Registry-National Institutes of Health
LAD: Left anterior descending coronary artery
LCX: Left circumflex coronary artery
LMCA: Left main coronary artery
LVEF: Left ventricular ejection fraction
MACCE: Major adverse cardiac and cerebrovascular event
MI: Myocardial infarction
MVD: Multivessel disease
NFMI: Non-fatal myocardial infarction
NSTEMI: Non-ST-segment elevation myocardial infarction
PCI: Percutaneous coronary intervention
PY: Pack year
RCA: Right coronary artery
S2DT: Symptom-to-door time
STEMI: ST-segment elevation myocardial infarction
TIMI: Thrombolysis In Myocardial Infarction
TIT: Total ischemic time.
