# Supplementary material for: Association between baseline smoking status and clinical outcomes following myocardial infarction
Source: Front Cardiovasc Med. 2022 Jul 22;9:918033. doi: 10.3389/fcvm.2022.918033 (PMC9354586; doi:10.3389/fcvm.2022.918033)
Supplement: Supplementary file 1 [file Table_1.DOCX]

**Table S1.** Baseline characteristics of a total of 503 patients who deceased during the initial hospitalization

| Characteristics | Frequency  (n = 503) | Characteristics | Frequency  (n = 503) |
| --- | --- | --- | --- |
| Smoking status |  | **Procedural characteristics** |  |
| Smokers | 209 (43.9) | **Use of PCI** | 388 (77.1) |
| <20 PYs | 47 (22.5) | **Femoral approach** | 335 (86.3) |
| 20-40 PYs | 85 (40.7) | **GPIIb/IIIa inhibitors** | 89 (22.9) |
| ≥40 PYs | 77 (36.8) | **Thrombus aspiration** | 106 (27.3) |
| Non-smokers | 267 (56.1) | **Image-guided PCI** | 37 (9.5) |
| Male patients | 322 (64.0) | **Infarct-related artery** |  |
| Age ≥75 years | 239 (47.5) | **LMCA or LAD** | 234 (60.0) |
| EMS utilization | 122 (24.3) | **Others (LCX or RCA)** | 156 (40.0) |
| Killip functional class III–IV | 316 (62.8) | **ACC/AHA lesion B2/C** | 345 (88.5) |
| BMI ≥25 kg/m^2^ | 96 (24.7) | **Preprocedural TIMI flow 0–I** | 278 (71.3) |
| Previous history |  | **LMCA disease** | 85 (18.5) |
| Hypertension | 305 (60.6) | **Multivessel disease** | 299 (65.0) |
| Diabetes mellitus | 204 (40.6) | **Prescribed medications** |  |
| Dyslipidemia | 31 (6.2) | **Aspirin** | 474 (94.2) |
| Prior IHD | 103 (20.5) | **P2Y12 inhibitors** | 463 (92.0) |
| Prior heart failure | 22 (4.4) | **Beta-blockers** | 47 (9.3) |
| Prior CVA | 70 (14.0) | **ACE inhibitors or ARBs** | 39 (7.8) |
| Family history of IHD | 14 (2.9) | **Statins** | 76 (15.1) |
| Serum creatinine ≥1.5 mg/dL | 205 (40.9) | **Use of thrombolysis** | 5 (1.0) |
| LVEF <40% | 144 (57.1) | **STEMI as a final diagnosis** | 328 (65.2) |

Values are presented as percentage (number) for categorical values.

ACC, the American College of Cardiology; ACE, angiotensin-converting enzyme; AHA, American Heart Association; ARB, angiotensin receptor blocker; BMI, body-mass index; CVA, cerebrovascular accident; EMS, emergency medical service; GPIIb/IIIa, glycoprotein IIb/IIIa; IHD, ischemic heart disease; LAD, left anterior descending coronary artery; LCX, left circumflex coronary artery; LMCA, left main coronary artery; LVEF, left ventricular ejection fraction; PCI, percutaneous coronary intervention; RCA, right coronary artery; STEMI, ST-segment elevation myocardial infarction; TIMI, Thrombolysis In Myocardial Infarction.
