# Supplementary figures and images for: Association between baseline smoking status and clinical outcomes following myocardial infarction
Source: Front Cardiovasc Med. 2022 Jul 22;9:918033. doi: 10.3389/fcvm.2022.918033 (PMC9354586; doi:10.3389/fcvm.2022.918033)

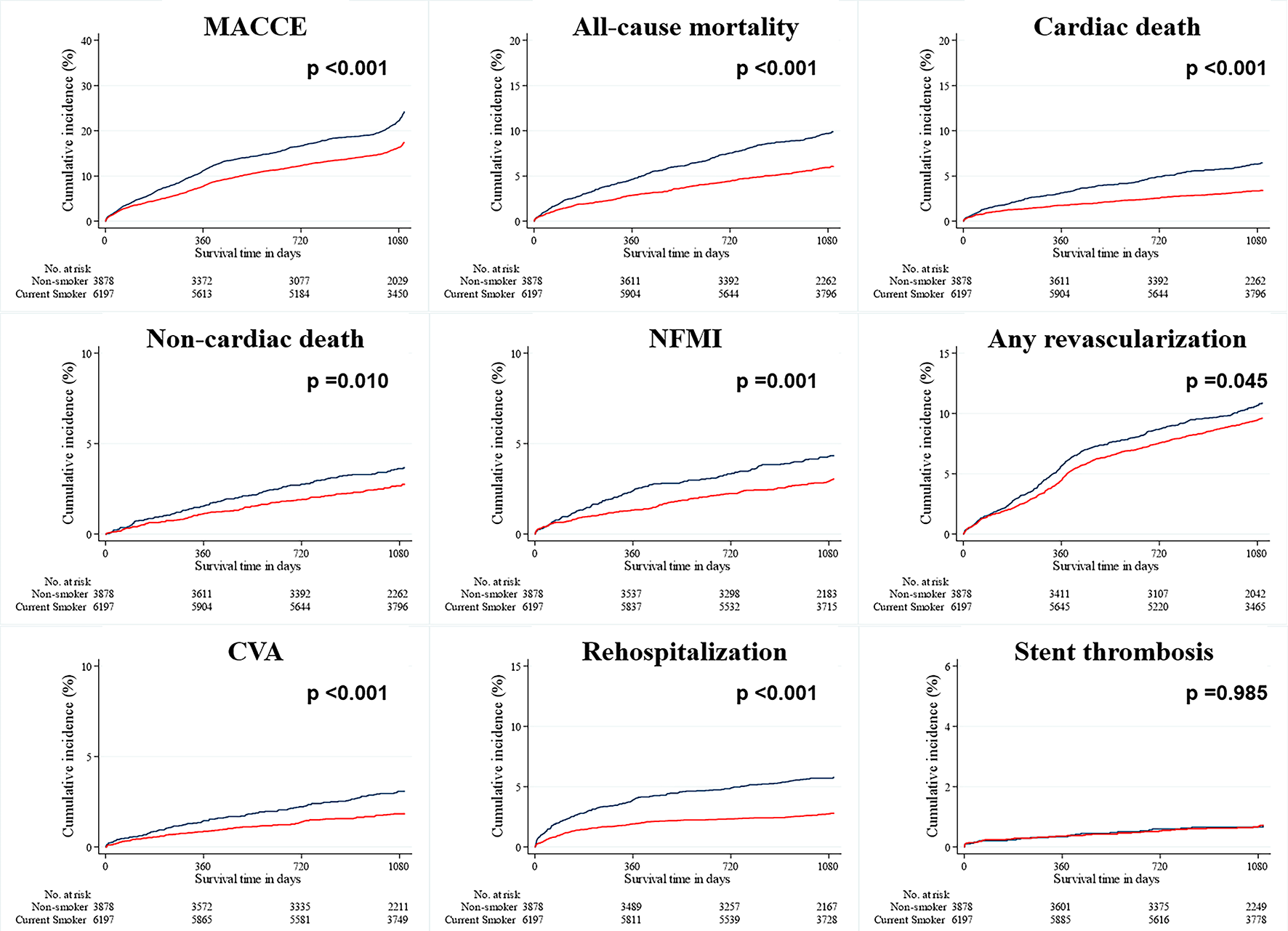

Supplement: Supplementary file 2 [file Image_1.TIF]

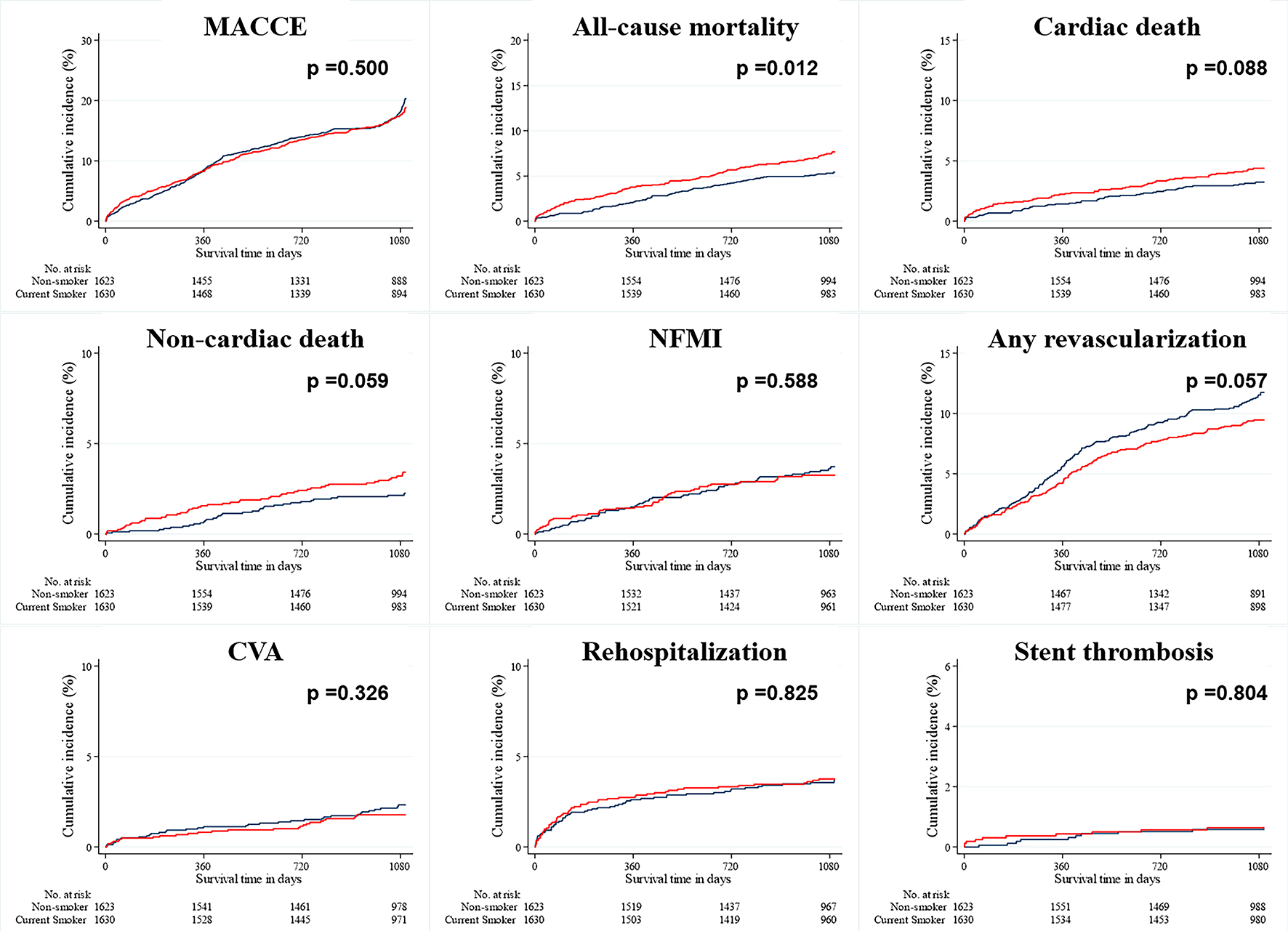

Supplement: Supplementary file 3 [file Image_2.TIF]
